# Management of Lateral Multiple-Level Metastasis in N1b Papillary Thyroid Microcarcinoma

**DOI:** 10.3389/fonc.2020.01586

**Published:** 2020-08-28

**Authors:** Wenlong Wang, Zhejia Zhang, Yunzhe Zhao, Wenbo Xue, Fadao Xia, Xinying Li

**Affiliations:** ^1^Division of Thyroid Surgery, Department of General Surgery, Xiangya Hospital, Central South University, Changsha, China; ^2^National Clinical Research Center for Geriatric Disorders, Xiangya Hospital, Central South University, Changsha, China

**Keywords:** lateral neck dissection, papillary thyroid microcarcinoma, extrathyroidal extension, predictive factor, recurrence

## Abstract

**Background:** The optimal extent of therapeutic lateral neck dissection (LND) in the management of N1b papillary thyroid microcarcinoma (PTMC) is still under debate in clinical practice. In this light, our study aims to explore the incidence, patterns, and predictive factors of the lateral multiple-level metastasis in N1b PTMC patients.

**Methods:** The clinical records of 142 patients diagnosed with N1b PTMC who underwent therapeutic LND from July 2015 to November 2018 at our institution were retrospectively reviewed. Univariate and multivariate analyses were conducted to examine the predictive factors associated with lateral multiple-level metastasis. The recurrence-free survival was analyzed and confirmed by Kaplan–Meier plots and log-rank test.

**Results:** The overall frequency of lateral multiple-level metastasis was 50.7% in N1b PTMC patients, and two-level to four-level simultaneous metastasis were present in 26.8, 17.6, and 6.3% patients, respectively. Extrathyroidal extension (ETE) (OR = 5.79, 95% CI, 1.36–24.59; *P* = 0.017) and the central metastatic lymph node ratio (CLNR) with values equal or higher than 0.61 (OR = 6.18, 95% CI, 2.53–15.09; *P* < 0.001) served as independent predictors of multiple-level metastasis in N1b PTMC patients. Moreover, locoregional recurrence was significantly higher in the selective neck dissection (SND) group compared to the modified radical neck dissection (MRND) one (HR = 3.65, 95% CI, 1.11–12.00; *P* = 0.03).

**Conclusion:** Our results show that the lateral multiple-level metastasis was relatively common, and we suggest MRND to be considered for N1b PTMC patients with ETE or CLNR equal or higher than 0.61.

## Introduction

Papillary thyroid microcarcinoma (PTMC) generally presents an indolent disease course with a good outcome. Recently, its incidence worldwide has increased dramatically, largely due to the development of high-resolution ultrasonography and improved health awareness (1–3). However, some PTMCs show aggressive behavior, such as gross extrathyroidal extension (ETE) and/or multiple-level metastasis at initial diagnosis, which indicates a high risk of locoregional recurrence and poor disease-free survival (3, 4). Several studies (5–7) have reported that the lateral neck metastasis in PTMC patients is a predictive factor for locoregional recurrence, thus further suggesting the need for the application of more aggressive treatments in patients with lateral lymph node metastasis (LNM).

The 2015 American Thyroid Association (ATA) consensus (8) recommended therapeutic lateral neck dissection (LND) for N1b PTMC patients; however, the optimal extent of therapeutic LND remained controversial. Data show that the conservative selective neck dissection (SND) increases the incidence of locoregional recurrence, whereas the radical LND causes potential complications associated with surgery (9–11). Therefore, an effective and appropriate surgical scope of LND proves crucial for patient survival.

The risk factors for the lateral LNM in PTMC patients have been investigated (12–15). To the best of our knowledge, though, lateral multiple-level metastasis in N1b PTMC patients has never been previously reported. Here, we focus our studies on some clinicopathological features of N1b PTMC patients. To better understand the behavior of lateral multiple-level metastasis, we have explored its patterns and clinical characteristics. The risk factors were assessed by studying the predictive factors of lateral multiple-level metastasis, whereas, to determine the rational extent of therapeutic LND in N1b PMTC, we compared the recurrence-free survival (RFS) rate between the modified radical neck dissection (MRND) and SND groups.

## Materials and Methods

### Patient Selection

A retrospective study was conducted for N1b PTMC patients who underwent therapeutic LND from July 2015 to November 2018 at our institution. A total of 142 patients were enrolled in the study, Physical examination, ultrasound (US), enhanced computed tomography (CT), and fine needle aspiration biopsy (*FNAB*) were routinely performed on all patients to assess the cervical lymph nodes and thyroid nodules before surgery. The study was approved by the Ethics Committee at Xiangya Hospital.

Patients were divided into two groups: the selective neck dissection (SND) group and a modified radical neck dissection (MRND) one. The MRND group included patients with LNDs classified with levels II to V, all characterized with preservation of the sternocleidomastoid muscle, internal jugular vein, and spinal accessory nerve. The SND group comprised patients with excision of the lateral neck lymph nodes with sparing one or more of the lateral neck levels (9, 16, 17). The inclusion criteria comprised all N1b PTMC patients who underwent total thyroidectomy with central neck dissection and therapeutic LND and had confirmed lateral LNM on histopathological examination. The exclusion criteria included incomplete medical records, history of previous thyroidectomy, distant metastasis, other histological types of thyroid cancer (follicular, medullary, and anaplastic), mixed type PTMC, or a follow-up time of less than 15 months. No robotic or endoscopic thyroidectomy were conducted on the enrolled patients.

### Surgical Strategy

**FIGURE 1:**
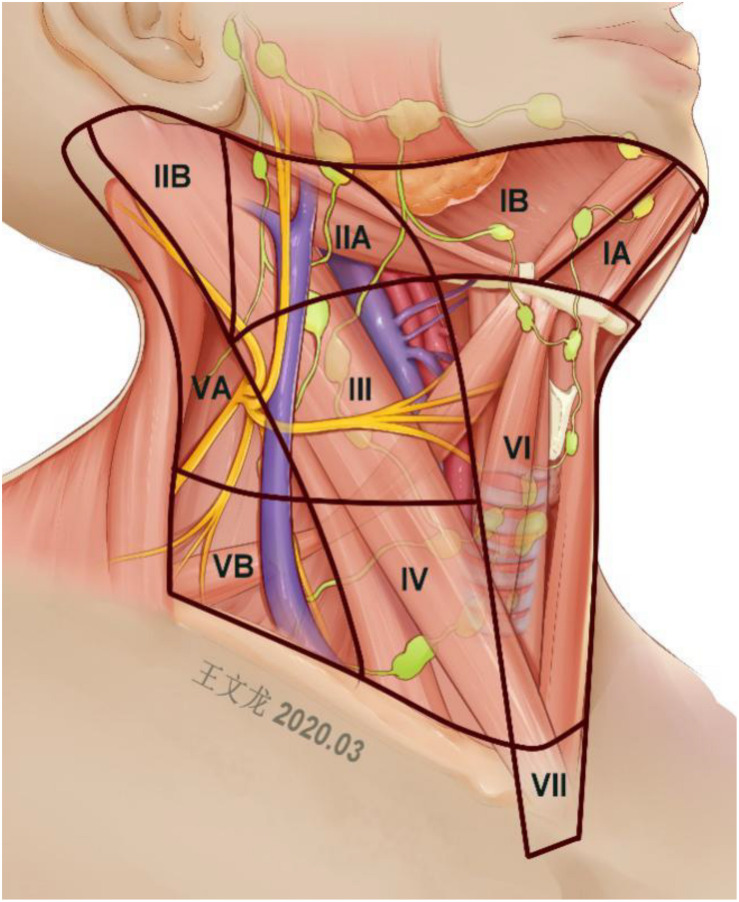
Anatomic levels of the neck dissection.

All surgeries were conducted at Xiangya Hospital in China. The exact extent of the neck dissection was decided mainly based on the intraoperative lateral neck lymph node status and the preoperative imaging examination. During the therapeutic LND, the LND levels were classified from I to V following the defined anatomic boundaries. Level I represented the submental and submandibular nodes, between the hyoid bone above and inferior to the posterior edge of the submandibular gland. Level II nodes were the upper jugular, spanning above the base of the skull and anterior to the border of the hyoid bone. Level III nodes were the mid- jugular ones. These lymph nodes were those bounded inferiorly by the cricoid cartilage and superiorly by the level of the hyoid bone. Level IV nodes were designated lower jugular and spanned the clavicle and the inferior border of the cricoid cartilage, whereas level V nodes comprised those in the posterior triangle, extending over the convergence of the lateral edge of the sternocleidomastoid muscle and the trapezius muscles to the clavicle (8, 18). Levels V, II, and I were further subdivided into sublevels as described in Figure 1. The thyroid surgeons separated the specimens according to the neck regions they were dissected from and sent the histological material to the Department of Pathology.

### Postoperative Follow-Up and Management

Thyroid-stimulating hormone (TSH) suppression therapy with or without radioactive iodine 131 was conducted after the initial surgery. In this study, all patients received standard follow-up within the first month and then every 3 months for the first year. Clinical evaluations such as physical examination, ultrasonography (US), blood levels of TSH, free thyroxine (FT4), free triiodothyronine (FT3), and thyroglobulin (TG) were measured at every patient visit. The long-term follow-up duration was for more than 15 months. Recurrence, withdrawal, and loss to follow-up were considered from the date of the last follow-up. Locoregional recurrence 15 months after the initial surgery was defined as the presence of metastatic lymph node by FNAB or histopathological examination.

### Statistical Analysis

Statistical analysis was performed using SPSS (22.0 version). Categorical variables were analyzed using Chi-squared and Fisher’s exact test. The Student’s *t*-test was used for continuous variables. The central metastatic lymph node ratio (CLNR) was defined as the number of central positive lymph nodes relative to the central total number of the harvested lymph nodes. Continuous variables were turned into categorical variables using the cut-off value that was calculated by receiver operating characteristic curve (ROC) analysis. Multivariate logistic regression analysis was conducted for the investigation of the predictive factors associated with the lateral multiple-level metastasis. Recurrence free survival (RFS) curves were drawn using Kaplan-Meier methods and were statistically analyzed using the log-rank test. *P*-values of less than 0.05 were considered statistically significant.

## Results

### Demographic and Clinicopathological Characteristics

A total of 142 N1b PTMC patients were selected (Table 1). Among them, 53 (37.3%) were men and 89 (62.57%) were women. The mean age was 38.06 ± 11.32 years. Only nine patients (6.3%) were older than 55 years. Among all cases, 42 (29.6%) were multifocal, 79 (55.6%) of all exhibited bilateralism, while 28 (39.4%) patients had evidence of Hashimoto’s thyroiditis (HT). The ETE was detected in 18 patients who accounted for 12.7%, while the capsule invasion was detected in 21 patients (14.8%). In 72.5% of all patients, the mean primary tumor size was measured as 0.71 ± 0.36 cm, whereas the estimated primary tumor size was larger than 0.5 cm. The calculated mean number of the central positive lymph nodes (CPLN) was 4.20 ± 3.75 and the mean central metastatic lymph node ratio (CLNR) was 0.41 ± 0.31. Skip metastases, defined as lateral LNM without the involvement of central LNM, were found in 23 patients (16.2%). SLD was performed in half of the patients (59.2%), whereas MRND was performed in 58 patients (40.8%). A total of 72 patients (50.7%) suffered from lateral multiple-level metastasis.

**TABLE 1:** Clinicopathological characteristics of PTMC patients (*n* = 142).

Characteristics	No. of patients
**Sex**	
Male	53 (37.3%)
Female	89 (62.7%)
**Age**	
**≥**55 years	9 (6.3%)
<55 years	133 (93.7%)
Mean ± SD	38.06 ± 11.32
Multifocality	42 (29.6%)
Bilateralism	79 (55.6%)
HT	28 (39.4%)
**ETE**	
Yes	18 (12.7%)
No	124 (87.3%)
Capsule invasion	21 (14.8%)
**Primary tumor size**	
≥5 mm	103 (72.5%)
<5 mm	39 (27.5%)
Mean ± SD	0.71 ± 0.36
Skip metastasis	23 (16.2%)
Central neck LNM	119 (83.8%)
CPLN (mean ± SD)	4.20 ± 3.75
CLNR (mean ± SD)	0.57 ± 0.35
**Lateral LNM**	
Multiple-level	72 (50.7%)
Single-level	70 (49.3%)
**Lateral neck dissection**	
MRND	58 (40.8%)
SND	84 (59.2%)
**Lateral neck recurrence**	
MRND group	3 (2.11%)
SND group	9 (6.34%)

*HT, Hashimoto’s thyroiditis; ETE, extrathyroidal extension; SD, standard deviation; LNM, lymph node metastasis; CPLN, central positive lymph nodes; CLNR, central metastatic lymph node ratio; SND, selective neck dissection; MRND, modified radical neck dissection.*

### Distribution of Lateral Multiple-Level Metastasis

**FIGURE 2:**
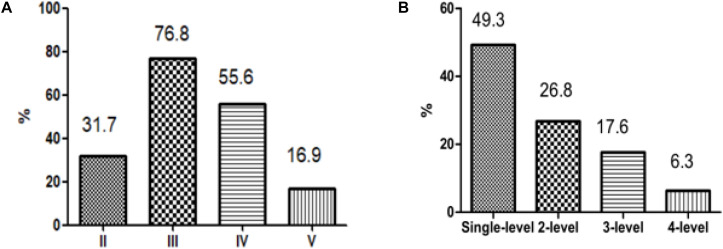
Patterns of metastasis. **(A)** According to the classification of the lateral neck dissection level. **(B)** According to the number of levels involved.

**FIGURE 3:**
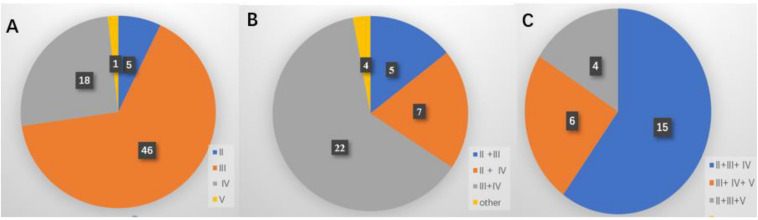
Distribution of the lateral neck lymph node metastasis. **(A)** Single-level metastasis. **(B)** Two-level metastasis. **(C)** Three-level metastasis.

Among all patients, level III metastasis was the most common and accounted for 109 patients (76.8%), followed by level IV found in 79 patients (55.6%), level II discovered in 45 patients (31.7%), and level V detected in 24 patients (16.9%) (Figure 2A). The single-, two-, three-, and four-level simultaneous metastases were detected in 70 (49.3%), 38 (26.8%), 25 (17.6%), and nine (6.3%) patients, respectively (Figure 2B). Of all 70 patients with the single-level metastasis, five (7.1%), 46 (65.7%), 18 (25.7%), and one (1.4%) had metastatic lymph nodes classified with II, III, IV, and V levels, respectively. Among the patients with the two-level metastasis 5 (13.2%), 7 (18.4%), 22 (57.9%), and 4 (10.5%) had simultaneous metastatic lymph nodes in II + III, II + IV, III + IV, and other levels, respectively. The patients with the three-level metastasis were presented with II + III + IV multiple-level of simultaneous metastases. They were diagnosed in 15 patients (60.0%), followed by multiple-levels III + IV + V, diagnosed in six patients (24.0%), and multiple-levels II + III + V, found in four patients (16.0%), respectively (Figure 3).

### Predictive Factors for Lateral Multiple-Level Metastasis

The CLNR and CPLN were significantly lower in the absence of lateral multiple-metastasis patients compared with the presence of multiple-metastasis patients (2.53 ± 2.34 vs. 5.83 ± 4.14 and 0.41 ± 0.34 vs. 0.73 ± 0.29), and all *P*-values were <0.001. To determine the optimal cut-off values for the CLNR and CPLN, ROC analysis was performed. The area under the ROC curve (AUC) was 0.753 and 0.671 (all *P*-values were <0.05), and the optimal cut-off values of CLNR and CPLN were 0.61 and 4.5, respectively. Therefore, patients were divided into two groups according to the CLNR and CPLN. The first group encompassed patients with CLNR and CPLN values ≥0.61 and ≥5, respectively. The second gathered patients with CLNR and CPLN values <0.61 and <5, respectively. In the univariate analysis, the lateral multiple-level metastasis was significantly associated with multifocality (*P* = 0.014), ETE (*P* = 0.003), CPLN ≥ 5 (*P* < 0.001) and CLNR ≥ 0.61 (*P* < 0.001). In the multivariate analysis ETE (OR = 5.79, 95% CI, 1.36–24.59; *P* = 0.017) and CLNR ≥ 0.61 (OR = 6.18, 95% CI, 2.53–15.09; *P* < 0.001), these served as independent predictors of lateral multiple-level metastasis in N1b PTMC patients (Tables 2, 3).

**TABLE 2:** Univariate analysis of risk factors of lateral multiple-level metastasis in PTMC patients.

Variables	Multiple-level metastasis			
	Present (*n* = 72)	Absent (*n* = 70)	X^2^/t	*P*
Sex			0.153	0.696
Male	28 (38.9%)	25 (35.7%)		
Female	44 (61.1%)	45 (64.3%)		
Age			0.151	0.698
≥55 years	4 (5.65)	5 (7.1%)		
<55 years	68 (94.4%)	65 (92.9%)		
Mean ± SD	36.26 ± 11.55	39.91 ± 10.84	1.940	0.054
Multifocality			6.080	0.014
Yes	28 (38.9%)	14 (20.2%)		
No	44 (61.1%)	56 (80.0%)		
Bilateralism			2.642	0.267
Yes	41 (56.9%)	38 (54.3%)		
No	31 (43.1%)	32 (45.7%)		
HT			0.115	0.735
Yes	15 (20.8%)	13 (18.6%)		
No	57 (79.2%)	57 (81.4%)		
ETE			8.780	0.003
Yes	15 (20.8%)	3 (4.3%)		
No	57 (79.2%)	67 (95.7%)		
Capsule invasion			0.094	0.759
Yes	10 (13.9%)	11 (15.7%)		
No	62 (86.1%)	59 (84.3%)		
Primary tumor size			2.015	0.156
≥5 mm	56 (77.8%)	47 (67.1%)		
<5 mm	16 (22.2%)	23 (32.95%)		
Mean ± SD	0.74 ± 0.32	0.69±0.39	0.853	0.395
CPLN			17.419	<0.001
≥5	40 (55.6%)	15 (21.4%)		
<5	32 (44.4%)	55 (78.6%)		
CLNR			36.486	<0.001
≥0.61	55 (76.4%)	18 (25.7%)		
<0.61	17 (23.6%)	52 (74.3%)		

*HT, Hashimoto’s thyroiditis; ETE, extrathyroidal extension; SD, standard deviation; LNM, lymph node metastasis; CPLN, central positive lymph nodes; CLNR, central metastatic lymph node ratio.*

**TABLE 3:** Predictive factors of lateral multiple-level metastasis in PTMC patients.

Variables	OR (95% CI)	*P*
Multifocality	2.22 (0.92–5.37)	0.077
ETE	5.79 (1.36–24.59)	0.017
CPLN ≥ 5	1.88 (0.74–4.76)	0.18
CLNR ≥ 0.61	6.18 (2.53–15.09)	<0.001

*ETE, extrathyroidal extension; LNM, lymph node metastasis; CPLN, central positive lymph nodes; CLNR, central metastatic lymph node ratio.*

### Prognostic Impact of Therapeutic LND on Locoregional Recurrence

Our results showed that 12 patients had developed locoregional recurrence, while no patient had distant metastasis nor died during the follow-up. Locoregional recurrence was detected in nine (6.34%) patients from the SND group and three patients in the MRND group (2.11%). The log-rank test showed that the locoregional recurrence was significantly higher in the SND group compared with the MRND group (HR = 3.65, 95% CI, 1.11–12.00; *P* = 0.03) (Figure 3). Among 12 patients, two (16.7%) exhibited recurrence at the central compartment, four (33.3%) in the contralateral lateral compartment, and six (50.0%) in the ipsilateral lateral compartment. In 10 patients with lateral neck recurrence, three recurred at level II and two at level III. One patient recurred at level V, whereas two did at levels III and IV, one at levels II and IV, and one at levels II and V.

## Discussion

An ongoing debate concerns the optimal extent of therapeutic LND in the management of N1b PTMC patients (2, 19–21). Considering the excellent prognosis and potential complications associated with surgery, the extensive LND is not well recognized nor yet accepted in the clinical practice. Several studies (22, 23) recommend conservative LND for N1b PTMC; however, it may increase the regional recurrence rate and decrease the disease-free survival. Therefore, decisions regarding a rational extent of the therapeutic LND should be considering the balance between the functional outcome and the oncologic safety. To the best of our knowledge, this is the first study that investigates the surgical scope of LND in PTMC patients with lateral multiple-level metastasis.

Previous studies (24, 25) have reported that the rate of lateral multiple-level metastasis after MRND in N1b papillary thyroid carcinoma (PTC) patients was greater than 70%. However, we found that the overall incidence of lateral multiple-level metastasis was 50.7% in N1b PTMC patients, which is partly due to the possibility of occurrence of the lateral LNM in PTMC patients, and 70 N1b PTMC patients (49.3%) underwent conservative SND in our study. The overall incidence of metastasis in levels II, III, IV, and V was 31.7, 76.8, 55.6, and 16.9%, respectively. Levels III and IV were the highest risk sites of lateral LNM. We have shown that the occurrence of levels III and IV can be treated as a recommendation for the subsequent dissection in N1b PTMC patients, which is similar to previous studies (1, 15, 25). Besides, our study further examined the pattern of lateral multiple-level metastases and found that among 72 PTMC patients with lateral multiple-level metastases, the two-, three-, and four-level simultaneous metastases were detected in 38, 25, and nine patients, respectively. Levels III + IV simultaneous metastases were the most frequently involved in the lateral two-level metastases, which was consistent with the anatomy of lymphatic drainage in the area of the thyroid (26). The three-level metastases including levels II, III, and IV were the most common patterns in N1b PTMC patients. Other authors (27) have also found that in the anterolateral groups (levels II, III, and IV) were the most frequently involved in the multiple-level node disease.

We therefore further evaluated the predictive factors for the lateral multiple-metastasis in N1b PTMC. Other studies (4, 14, 28) have reported that CPLN was a predictor of lateral LNM in PTC patients. Xiao et al. (29) conducted ipsilateral LND for PTC patients and demonstrated that CPLN ≥ 2 was a reliable indicator of lateral LNM. Likhterov and colleagues (26) revealed that the incidence of lateral LNM significantly increased with the CPLN. Kim et al. (24) have reviewed 658 N1b PTC patients and demonstrated that the bilateral central LNM (OR = 4.06, 95% CI: 2.29–7.18) and the unilateral central LNM (OR = 2.45, 95% CI: 1.53–3.92) were independent predictors for the lateral multilevel metastasis. In our research, the CPLN in patients with lateral multiple-metastasis was significantly higher than in patients where the lateral multiple-metastasis was absent (5.83 ± 4.14 vs. 2.53 ± 2.34, *P* < 0.001), and, to elucidate the quantitative relationship between the lateral and central LNM, we used the ROC curve analysis that revealed the optimal cut-off values of CPLN was 4.5, while CPLN values ≥ 5 were significantly associated with lateral multiple-level metastasis (*P* < 0.001), which is in accordance with Bohec’s study (30). However, in multivariate analyses, CPLN values ≥5 cannot be of value in predicting lateral multiple-level metastases (*P* = 0.18). Importantly, only ETE (OR = 0.79, *P* = 0.017) and CLNR (OR = 6.18, *P* < 0.001) were independent predictors for lateral multiple metastasis in N1b PTMC patients. Therefore, we suggest that a more extensive dissection is necessary for N1b PTMC patients with ETE or CLNR ≥ 0.61.

**FIGURE 4:**
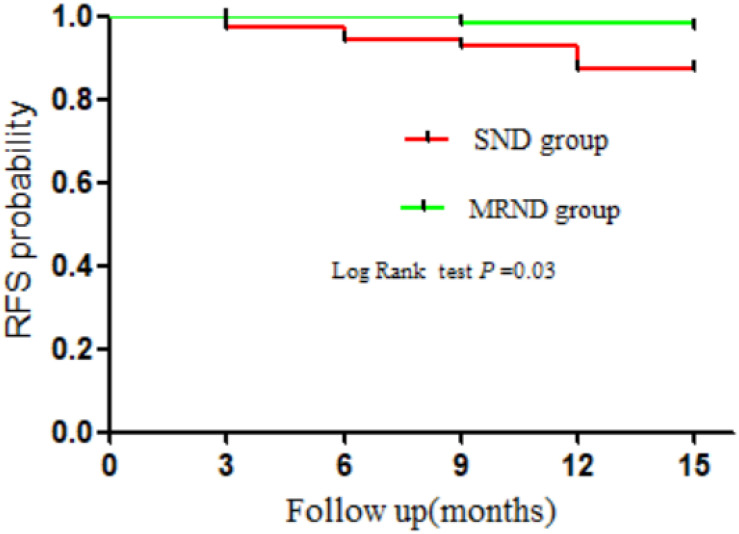
Kaplan-Meier plots of recurrence-free survival (RFS) rates for N1b PTMC patients. SND, selective neck dissection; MRND, modified radical neck dissection.

Next, we explored the prognostic impact of therapeutic LND on locoregional recurrence. Many studies have reported the association between the recurrence risk and the extent of LND, but the exact extent of the LND from a prophylactic LND to a comprehensive neck dissection of all lateral neck lymph nodes have not been well understood (11, 23, 31, 32). A systematic review and meta-analysis (19) have revealed that the extent of LND may not change the rate of recurrence and recommended SND for N1b PTC patients without any other risk factors. Kobayashi et al. (10) also believed that the conservative SND may represent a suitable treatment for patients at low-risk. However, Strajina et al. (17) conducted a-single center study over 10 years and demonstrated that a more extensive dissection could decrease the rate of lateral neck recurrence after LND for PTC patients. In our study, the log-rank test showed that SND significantly increased the risk of RFS rates in N1b PTMC patients (HR = 3.654, 95% CI: 1.11–12.00, *P* = 0.03) (Figure 4). Therefore, more extensive dissection is necessary for PTMC patients with lateral multiple metastasis.

However, our study has some limitations. First, this is a single-center retrospective study, the level of evidence was weaker than any other prospective and multicenter study. Second, for 124 N1b PTMC patients with prognostic analysis, there was no information about the radioiodine therapy (RAI), and the follow-up periods were too short to accurately assess the RFS rate. This affects the related results. Third, the complications associated with MRND and SND including wound infection, shoulder syndrome, hemorrhage and chylous leakage had not been recorded in our medical system. However, our study collected data from a single center and used strict inclusion/exclusion criteria presenting a reliable and accurate result in the rational management of LND in the treatment for PMTC patients with lateral multiple-metastasis, which has not been reported before.

## Conclusion

In conclusion, the incidence of the lateral multiple-level metastasis has been common among all studied patients. The meticulous evaluation of the lateral neck proves important for N1b PTMC patients with ETE or CLNR with values equal or higher than 0.61 and serves as a recommendation for subsequent more extensive dissection decision.

## Data Availability Statement

The raw data supporting the conclusion of this article will be made available by the authors, without undue reservation.

## Ethics Statement

The studies involving human participants were reviewed and approved by the Ethics Committee of Xiangya Hospital. The patients/participants provided their written informed consent to participate in this study.

## Author Contributions

WW, ZZ, YZ, WX, and XL: data collection and analysis, and manuscript review. WW, FX, and XL: writing-original draft and manuscript preparation. WW and XL: writing the manuscript and methodology. All authors: results interpretations.

## Conflict of Interest

The authors declare that the research was conducted in the absence of any commercial or financial relationships that could be construed as a potential conflict of interest.
